# Rural Missourians’ perspectives on pain: “I like to be in control of my life”

**DOI:** 10.1016/j.healthplace.2025.103561

**Published:** 2025-10-14

**Authors:** Karla T. Washington, Klaudia Kukulka, Archana Bharadwaj, Olivia J. Landon, Masako Mayahara, Jacquelyn J. Benson

**Affiliations:** a Department of Medicine, Washington University School of Medicine, 4590 Nash Way, St. Louis, MO, 63110, USA; b School of Medicine, University of Missouri, 1 Hospital Dr., Columbia, MO, USA; c Department of Anesthesiology, Washington University School of Medicine, 660 S Euclid Ave., St. Louis, MO 63110, USA; d Center for Public Partnerships and Research, University of Kansas, 1617 St. Andrews Dr., Lawrence, KS, 66047, USA; e Goldfarb School of Nursing at Barnes-Jewish College, 4483 Duncan Ave., St. Louis, MO, 63110, USA

**Keywords:** Culture, Non-pharmacological, Pain management, Pharmacological, Place, Qualitative, Rural

## Abstract

This study examined rural Missourians’ conceptualization of pain and their attitudes toward pharmacological and non-pharmacological pain management strategies. The study sample consisted of twenty-five (N = 25) community-dwelling adults residing in rural Missouri counties. Researchers conducted semi-structured qualitative interviews and applied thematic analysis techniques to interpret the data. Results indicated that participants often viewed pain as a limitation and associated its experience and treatment with weakness. Their attitudes toward pharmacological pain management were influenced by a prevalent social stigma surrounding pain medications (particularly opioid analgesics), fear of losing control, and a general aversion to medications. In contrast, their attitudes toward non-pharmacological pain management were decidedly positive. Participants expressed a strong preference for natural interventions and emphasized preventive measures to manage pain. Study findings support previously published research suggesting that rural individuals may minimize medical interventions and prioritize self-sufficiency. To address these cultural norms effectively, a strong clinician-patient relationship and multimodal pain management approaches that incorporate non-opioid strategies are recommended.

## 1. Introduction

For decades, pain has been a leading cause of patients seeking medical care in the United States (Healthcare Cost and Utilization Project, 2018). It is one of the most disabling, burdensome, and costly conditions in the nation (Davis et al., 2011). Although pain is often conceptualized primarily as a physical phenomenon, the International Association for the Study of Pain defines pain as “an unpleasant sensory and emotional experience associated with or resembling that associated with actual or potential tissue damage” (Raja et al., 2020). Indeed, a reciprocal relationship between the physical and emotional dimensions of pain has been well documented, in which pain exacerbates conditions such as depression and anxiety, and these conditions, in turn, intensify the experience of pain (Wasilewski et al., 2010; Griffin et al., 2022; Werneck and Stubbs, 2024).

In addition, a growing body of research highlights the influence of place in shaping perceptions and experiences of pain. This is particularly true in the context of rural communities. Extensive research demonstrates that individuals residing in rural areas bear a disproportionate burden of pain. Compared to their urban counterparts, rural residents are more likely to report pain (Tripp et al., 2006; Baker et al., 2024), describe it as more severe (Baker et al., 2024), and, during middle age, expect fewer pain-free years (Sun et al., 2024). In many rural settings, people living with pain are more likely to be prescribed opioid medication (Prunuske et al., 2014) and lack adequate access to non-pharmacological pain treatments, such as physical therapy (McLaughlin, 2025). Qualitative studies enrich this body of work by examining how culture, as embedded in place, shapes the ways pain is understood, expressed, and addressed in rural communities. These studies identify values such stoicism, dignity, and self-sufficiency as powerful influences on how rural residents communicate about and respond to pain and make decisions regarding its management, often leading to silent suffering and reluctance to utilize formal healthcare services (Tollefson et al., 2011; Kukulka et al., 2024).

### 1.1. Theoretical foundation

Exploration of the role of culture in the understanding and experience of pain is theoretically supported by Kleinman’s Explanatory Model of Illness, which posits that individuals interpret and respond to health conditions through culturally shaped beliefs, values, and experiences (Kleinman et al., 1978; Kleinman, 1980). The model emphasizes that people hold their own explanations for symptoms, including their causes, expected course, and appropriate treatments, which may differ from the perspectives of healthcare providers. In the context of pain, these culturally informed interpretations shape not only how individuals conceptualize pain but also the strategies they consider acceptable for managing it.

Applying this lens to rural communities reveals how place and culture intersect to shape understandings of pain. In these settings, local values, such as independence, self-sufficiency, and a strong work ethic, are reinforced by geographic isolation, limited healthcare access, and the need for often physically demanding work. (Holmes and Levy, 2015) Kleinman’s model suggests that these factors may influence how rural residents describe and respond to pain, as well as which pain management strategies they find acceptable. Thus, understanding pain within the context of rural places can help illuminate how environment, labor demands, and other place-based cultural expectations converge, illustrating the ways that lived experiences of pain are socially and geographically situated.

### 1.2. Rural Missouri

Located in the Midwestern United States, Missouri has a substantial rural population, with approximately 2.05 million rural residents comprising just over one-third of the total state population. (Missouri Department of Health & Senior Services, 2023) Approximately 90 % of rural Missourians are non-Hispanic White, and just over half report high school (39.5 %) or less (12.4 %) as their highest level of formal educational attainment. (Missouri Department of Health & Senior Services, 2023) Compared to urban residents, rural Missourians are more likely to live in poverty, particularly if they are a child or older adult, and to be medically uninsured. Access to healthcare is also limited, with some rural counties averaging nearly 30 miles to the nearest hospital. (Missouri Department of Health & Senior Services, 2023) However, rural Missourians benefit from a lower cost of living, more ready access to natural spaces, and a slower pace of life that can support well-being (Missouri Department of Health & Senior Services, 2010). They are also more likely than urban Missourians to have tightly knit social networks, generating bonding capital that enables groups, organizations, and communities to launch locally led economic, health, and community development efforts. (University of Missouri Extension, 2025)

Rural Missourians’ daily lives are strongly influenced by the state’s diverse geographic and economic contexts. (Missouri Department of Agriculture, 2025; Missouri Department of Conservation, 2025) Rural areas in the north and west are predominantly agricultural, characterized by extensive row crop (e.g., corn, soybeans) cultivation and livestock production. The southern Ozarks are defined by forested hills, karst topography, and river systems, with local economies supported by tourism, forestry, and small-scale agriculture. In the southeast, the Bootheel region (named for its resemblance to a boot heel on the state map) consists of fertile alluvial plains that sustain highly productive farmland, where cotton, rice, melons, and peanuts are grown. (Missouri Department of Agriculture, 2025; Missouri Department of Conservation, 2025) Collectively, these regions illustrate the environmental and economic heterogeneity that shapes rural Missouri life.

#### 1.2.1. Pain in rural Missouri

Risk factors for pain observed elsewhere in the United States also play a significant role in rural Missouri, which is characterized by an older population, lower socioeconomic status, more physically demanding jobs, and more formidable barriers to healthcare access than urban areas of the state. (Missouri Department of Health & Senior Services, 2023) Rural cultural ideals may also be relevant to the experience of pain among rural Missourians. For example, respect for toughness and viewing the body as a means to fulfill one’s responsibilities are common values among rural Missourians (Kukulka et al., 2024), which may contribute to feelings of shame and the experience of social stigma among pain sufferers. These perspectives, coupled with a statewide public health crisis of opioid use disorder (Grin, 2024), may explain documented instances of prejudice against pain patients, described as “lazy”, “drug seekers”, and “malingerers” by some rural Missouri healthcare providers (Christopher et al., 2015).

### 1.3. Study purpose

Understanding how rural culture shapes approaches to pain is critical for informing statewide health policies and practices aimed at addressing pain and related disparities in rural Missouri. Prior studies have highlighted values common in rural communities, such as independence, self-reliance, and financial prudence, that influence decision-making about health, including pain management. (Holmes and Levy, 2015) While rural culture is diverse and shaped by local contexts (Bennett et al., 2019), these values nonetheless contribute to distinctive ways that rural residents experience and respond to pain. To ensure that policy and practice initiatives in Missouri reflect these perspectives, we conducted a study to examine rural Missourians’ experiences and views. Our objective was twofold: 1) to understand how rural Missourians conceptualize pain, and 2) to explore rural Missourians’ attitudes toward pharmacological and non-pharmacological pain management.

## 2. Materials and methods

### 2.1. Participant recruitment

Our study was approved by the Institutional Review Boards (IRBs) at the University of Missouri and Washington University in St. Louis. We received support in recruiting study participants from two primary sources. First, we partnered with University of Missouri Extension, a statewide network with longstanding relationships and deep community ties across rural Missouri, which facilitated outreach to potential participants through its local offices. Second, we recruited through Washington University’s Volunteer for Health, a university-based research participant registry that disseminates study opportunities to individuals who have previously expressed interest in health-related research. Although its outreach extends beyond St. Louis, its urban location limits the depth of rural community engagement compared to University of Missouri Extension. Both recruitment partners distributed study flyers and shared informational announcements through email listservs and organization-affiliated social media platforms to broaden awareness of the study among potential participants.

We employed maximum variation purposeful sampling (Creswell and Poth, 2025), seeking broad representation of rural Missouri adults from across the state, consistent with our ultimate goal of informing statewide policy and practice initiatives. Individuals interested in learning more about research participation were instructed to contact our study coordinator, who followed up to provide additional information, perform study eligibility screening, and schedule subsequent research activities, including telephone interviews and compensation of $25 per participant. Inclusion criteria required that study participants were age 18 or older and resided in a rural Missouri county (defined as a county with a 2013 U.S. Department of Agriculture Rural-Urban Continuum Code greater than 3). (U.S. Department of Agriculture Economic Research Service, 2013) Participants included both individuals with and without chronic pain to capture a broad spectrum of attitudes toward pain, focusing on general perceptions rather than the experience of living with a chronic pain condition.

### 2.2. Data collection and analysis

We conducted audio-recorded, semi-structured, telephone interviews with all study participants (average interview duration = 62 min). The interview guide included a mix of open-ended questions (e.g., “Can you tell me about a time you were sick or hurt?”) and more directed prompts (e.g., “What is the first word that comes to mind when I say the word pain?”) to delve deeper into participants’ thoughts, feelings, and experiences. We also asked questions related to hypothetical scenarios. For example, we focused a series of questions around Tom, a (fictitious) 68-year-old retired man with terminal metastatic lung cancer that causes him significant pain. A professional third-party vendor transcribed all interviews in preparation for analysis.

Transcribed interview content was analyzed via inductive thematic analysis (Aronson, 1995). Each transcript was initially reviewed by at least two researchers, who used NVivo qualitative research software (Lumivero, Denver, CO) to independently apply descriptive labels to segments of data relevant to the study aim. These codes were generated inductively and iteratively, rather than drawn from a pre-determined codebook. Throughout this process, the researchers met regularly to discuss coding decisions, refine codes, and collaboratively develop an evolving set of candidate themes representing rural Missourians’ conceptualizations of pain and attitudes toward pain management. Candidate themes and supporting data segments were then discussed with the broader research team to further enhance the credibility (or “truth value”) and confirmability of our study findings (Henderson and Rheault, 2004).

## 3. Results

We analyzed interviews conducted with 25 rural Missourians to better understand how they conceptualized pain and to explore their attitudes toward pharmacological and non-pharmacological pain management (participant characteristics are summarized in Table 1). Thematic analysis resulted in the identification of seven themes, summarized in Table 2 and described in detail below along with illustrative quotations in which participants are referenced by numeric study identifiers (e.g., 107, 110, 121) to preserve confidentiality while allowing readers to distinguish individual perspectives. Each theme is described separately for clarity. However, they are not mutually exclusive, reflecting the complexity and, in certain instances, the interconnectedness of participants’ experiences and insights.

### 3.1. Conceptualizing pain

Two themes captured participants’ conceptualizations of pain: *pain as a limitation* and *pain as weakness*. When asked by their interviewer, “What is the first thing you think of when I say the word pain?” participants who conceptualized *pain as a limitation* answered with responses such as “an annoyance” or “a nuisance.” Participant 110 summarized many interviewees’ responses when describing pain as “something you don’t have time for,” while Participant 121 stated that they thought of pain as “interference with what [they] want to do.” Participant 107 stated, “It will be very distract[ing] for me [if] this pain is not going away. It will [...] not let me do my job productively.” These responses, which echo sentiments expressed throughout the dataset, captured participants’ experiences of pain as inhibiting productivity and limiting individual agency.

Participants also described *pain as weakness*, often equating pain tolerance with strength. Several participants echoed Participant 122’s assertion that “I am not a wimp,” seemingly taking pride in their ability to “handl[e] pain” (Participant 114). Participant 111 shared the following information: “I have a high tolerance to pain [...] I [once] walked on a broken ankle [...] I pretend like [the pain] is not there. I’m real good at that. I think you can make your mind believe anything if you work real hard at it.” Some participants attributed their pain tolerance to age and values instilled in them as a child. This was the case for Participant 117, who explained, “My generation was raised in a family that [if] you had something happen, you dealt with it, and you moved on [ .... ] I don’t think [the younger generations are] that strong.” Several participants shared stories suggesting that *pain as weakness* was a view shared by others in their family. For example, Participant 123, who reported experiencing chronic pain, recalled the following conversation with her father: “He sat me down and gave me this lecture and told me that if I didn’t start acting well and being healthy, my husband was going to leave me.” For many participants, viewing *pain as weakness* served as a deterrent to treating pain. This was true for Participant 114, who recalled often opting not to take pain medication due to their ability to “take quite a bit [of pain],” and Participant 119, who described being able to “make it through [pain]” without medication. Participants repeatedly emphasized the importance of toughness and described pain experiences and responding to those experiences in a manner intended to ameliorate (versus endure) pain as evidence of weakness.

### 3.2. Attitudes toward pharmacological pain management

Participants’ attitudes toward pharmacological pain management were reflected in three themes: *social stigma*, *fear of losing control*, and *general aversion to medication*. In numerous respects, participants’ awareness of *social stigma* was associated with their (previously described) conceptualization of *pain as weakness* (recall that interconnectedness of themes was an intended analytic outcome). The nuanced difference between the two themes was that data comprising the theme *social stigma* described a broader negative perception of pain sufferers, more specifically those who treated pain with opioid medications, that was perceived to be shared by the community at large. Alternately, views of *pain as weakness* were expressed or detected on the individual and/or familial level and were commonly associated with avoidance of all classes of pain medication, including non-opioid analgesics. Another difference was that the topic of *social stigma* was directly raised more often during interviews of participants who reported experiencing chronic pain and/or opioid analgesic use compared to those who did not, while descriptions of *pain as weakness* were broadly dispersed throughout the dataset.

Data segments describing the *social stigma* associated with pharmacological pain management were numerous. As one example, Participant 116 described an interaction with a pharmacist that they perceived to be rooted in negative assumptions about patients, like themselves, who were prescribed opioid medications: “I always get ‘the lecture.’ [A pharmacist once] tried to convince me to buy a [naloxone] kit in case I overdosed at home. It’s like, ‘No sir, I’ve been doing this for 30 years. I am a responsible person. I can be trusted.’” Similarly, Participant 123 explained, “The people who try to get help [for pain] are often shamed by the doctors, nurses, and family. [The implication is that] they should be able to handle this without medication.” Importantly, this *social stigma* did not typically extend to individuals prescribed medications to treat terminal pain (i.e., those “on their deathbed”). When presented with a hypothetical scenario of a terminally ill man experiencing pain, nearly all participants readily indicated that it would be deemed socially acceptable for him to take pain medications, including opioids. Participant 109 explained, “I’ve worked in a nursing home and watched people die. I do believe that there is an appropriate time sometimes [for opioids].”

On a more individual level, many participants’ attitudes toward pharmacological pain management were rooted in a *fear of losing control*. Two subthemes related to a broader *fear of losing control* were *fear of addiction* and *fear of incapacitation*. Regarding *fear of addiction*, Participant 124, like many other participants, shared that “get[ting] hooked on drugs [was] one of [their] big fears.” Similarly, Participant 114 stated, “I wouldn’t want to be a slave to anything.” Relatedly, several participants described widespread substance misuse in their communities, sometimes resulting in accidental overdose, which informed their *fear of addiction*. Many participants reported personally knowing someone who had experienced negative consequences associated with opioid misuse or addiction, as was true for Participant 126, who recalled the following: “A doctor and his wife lost their son to an opioid overdose. They’ve written a very eloquent piece on the dangers of opioid use [...] in the community [...] If it could be [their] kid, it can be anyone’s.”

Data comprising the subtheme *fear of incapacitation* were often co-coded with *fear of addiction*, as was the case for the following data segment, provided by Participant 121: “I am not taking anything that’s going to addict me and cause me to lose function.” *Fear of incapacitation* and *fear of addiction* were determined to be overlapping issues for Participant 118 as well. They stated, “I don’t like taking stuff that I don’t realize what it’s going to do to me. I like to be in control of my life.” However, for most participants, *fear of incapacitation* was a concern beyond addiction as well. These participants described an aversion to taking pain medications due to expectations about how the medications would cause them to feel, using words like “dulled”, “dimmed”, or “altered.” Participant 122 explained, “I don’t ever want to take something that’s going to make me to where I am mentally incapacitated. My mind is one of my strongest features.” Participant 103 similarly expressed, “I don’t like my mental clarity being affected. That’s the reason I don’t take [pain medications].” Participant 121 succinctly opined, “Quality of life means you have your brain.” A few participants described problematic prior use of pain medications. For example, Participant 105 shared, “I was taking [oxycodone] and anxiety pills, sleeping pills, muscle relaxants, too many ‘benzos.’ I just feel like I slept for 20 years.” A common element among these and all data segments comprising the subthemes *fear of addiction* and *fear of incapacitation* was a broader emphasis on the importance of self-control and, to the extent possible, control over one’s circumstances.

Participants’ generally negative perception of pharmacological pain management was also found to be rooted in a *general aversion to medication*. Multiple individuals expressed negative attitudes toward medication generally (e.g., “I just don’t like it” [Participant 117]) and toward pain medication more specifically (“It’s been at least five years since the last time I’ve had an ibuprofen” [Participant 101]). Data segments comprising this theme tended to be non-specific regarding the reasons for participants’ *general aversion to medication* (thus, the inclusion of the word “general” in the theme). In a few instances, participants provided additional insights. For example, several participants shared a general understanding that “no matter what medications you are taking, no matter how safe the medications are, some side effects can [always] occur” (Participant 107). However, when prompted to further discuss their *general aversion to medication,* a greater number of participants segued into a discussion of their preference for more “natural” approaches to healthcare, as described below in our presentation of results pertaining to participants’ attitudes toward non-pharmacological pain management.

### 3.3. Attitudes toward non-pharmacological pain management

Participants expressed decidedly positive attitudes toward non-pharmacological pain management. These attitudes were represented in two themes: *preference for natural interventions* and *emphasis on prevention*. Participants commonly described their preferred approach to pain management as “natural,” emphasizing a strong *preference for natural interventions*. Participant 107 discussed the value of yoga or stretching for lower back pain, while Participant 114 noted the need for “certain minerals and vitamins to be able to heal.” In addition, several participants recalled feeling surprised when they learned about relatively simple and effective non-pharmacological methods of pain control, such as ice (e.g., “I never realized something as simple as ice could bring such relief,” [Participant 106)]; “I didn’t think I would like [ice], but that really worked, and so I don’t think I took any narcotics after the meniscus surgery” [Participant 115]). Physical exercise, rest, deep breathing, guided imagery, and meditation were also cited as examples of preferred natural interventions. Participant 109 recommended that pain sufferers “not sit around. You’ve gotta get up and move that thing around [ .... ] Our body has a lot of natural pain killers,” while Participant 117 advocated for “self-meditation, not medication.” In addition, although few participants discussed marijuana use, those endorsing it as a pain management strategy explained that they viewed it as a “natural [...] alternative” to pain medications (Participant 122).

Although some “natural” approaches to pain management were initially labeled as “alternative” or “holistic,” subsequent analyses indicated that participants’ *preference for natural interventions* was not necessarily incompatible with conventional or mainstream healthcare. Interventions such as physical therapy were commonly suggested as favorable approaches to pain management (e.g., “Physical therapy could strengthen [...] muscles and stuff. That might help with [...] pain [Participant 125]), and several participants endorsed psychotherapy or professional counseling as a potentially efficacious pain management strategy that might “help [one] cope” with pain (Participant 106) and “stay mentally in the game” (Participant 103). Rather than reflecting a broader skepticism or disdain for formal healthcare, participants’ *preference for natural interventions* served as the affirmative counterpart to their *general aversion to medication*. That is, participants considered interventions natural if they provided an alternative to “all these [...] drugs and pills that I don’t even know the names of [Participant 122]).”

Participants also placed an *emphasis on prevention* of pain, which was typically thought to be achieved by regular physical activity (“If you don’t use it, you’re going to lose it” [Participant 115]) and “eating healthy” (Participant 110). Participant 107 explained that if “you don’t stay active, the muscle of your body will [...] shrivel,” and Participant 111 stated, “The more oxygen you get in your body, the better you are.” Other pain prevention recommendations included stretching, walking, cycling, and seeking professional support to manage stress. Along with their *preference for natural interventions*, participants’ *emphasis on prevention* underscored highly favorable attitudes toward non-pharmacological approaches to pain.

## 4. Discussion

In conducting this qualitative research study, we sought to better understand how rural Missourians conceptualize pain and how they view both pharmacological and non-pharmacological pain management, with an ultimate goal of informing culturally responsive pain management strategies for this and other medically underserved rural populations. We found that participants conceptualized pain as a limitation and often equated its experience and treatment as weakness. Participants’ attitudes toward pharmacological pain management were found to be informed by a widely regarded social stigma toward pain medications (particularly opioid analgesics), fear of losing control, and a general aversion to medications. However, participants described decidedly positive attitudes toward non-pharmacological pain management, voicing a strong preference for natural interventions and emphasizing pain prevention whenever possible.

In several aspects, our research findings heavily reflect recently described rural values embedded in self-sufficiency, respect for toughness and resilience, as well as the view of the human body as an instrument to fulfill one’s familial and social responsibilities (Kukulka et al., 2024). Furthermore, our research builds on the idea of rural individuals as medical minimizers (Groopman and Hartzband, 2012), or individuals who are likely to avoid medical intervention if they perceive it to be unnecessary. Individuals who exhibit medical minimization tendencies would likely express disagreement with statements such as, “When I feel unwell, my immediate response is to visit a doctor and obtain a prescription,” but they would likely agree with statements like, “If I have a health issue, my preference is to wait and see if the problem gets better on its own before going to the doctor.” (Scherer et al., 2016) The present study findings illustrate the application of medical minimization to pain treatment, as participants expressed pride in having a “high pain tolerance” and “handling pain” rather than appearing “weak” by using medication to control their pain.

Building from Kleinman’s Explanatory Model of Illness (Kleinman et al., 1978; Kleinman, 1980), our findings demonstrate how participants’ perceptions of pain and its management are deeply embedded in rural cultural and place-based contexts. Participants’ attitudes reflect not only individual and family-level beliefs but also broader community expectations about resilience, self-reliance, and acceptable responses to suffering. Connections to physical place are reflected in participants’ preferences for natural interventions, which align with rural residents’ ready access to outdoor and natural environments. Further, in rural contexts, rugged geography and physically demanding work, such as farming and forestry, require resilience and perseverance. From an early age, rural individuals may learn that enduring discomfort or injury is necessary to complete labor-intensive tasks and fulfill responsibilities tied to the land. These cultural values may persist even in rural Missouri communities with economies shifting away from agriculture into industries like manufacturing and tourism (Rural Local Initiatives Support Corporation (LISC and), 2023; Missouri Department of Economic Development, 2015), as they are often transmitted intergenerationally (Tam, 2015).

### 4.1. Practice implications

This study highlights a need for clinicians to be aware of place-based beliefs about pain and its management to develop acceptable and culturally responsive pain interventions. In this rural population, patients were reluctant to use pharmacological approaches, especially those involving opioids, to manage their pain and strongly preferred non-opioid pain management strategies. This belief lends itself well to multimodal pain management, defined as “the use of more than one medication or class of medication, or the use of more than one analgesic technique to produce analgesia through multiple mechanisms.” (Crews, 2002)

Initiatives can also be taken to support patients in rural communities in the application of home-based pain management strategies and corresponding use of coping skills before or, when medically appropriate, as an alternative to seeing a clinician. These measures are especially needed in the context of protracted medical appointment waiting times as well as lengthy commutes to healthcare facilities, as noted in prior research (Kukulka et al., 2024). By increasing patient awareness and confidence in using pain management techniques, such as the application of ice, which participants 106 and 115 found effective, through direct patient education or by collaborating with pain management specialists to develop web-based content or community-based outreach efforts, clinicians can support patients in pain self-management, which would likely be viewed as highly congruent with cultural emphases on patient autonomy and personal responsibility for one’s health. Importantly, however, these strategies should complement, not replace, efforts to address structural barriers and social determinants of health, including limited access to care, poverty and other socioeconomic constraints, and declining community resources, all of which profoundly influence pain management and broader issues pertaining to rural health equity (Ziller and Milkowski, 2020).

In addition, patients with pain management needs could be linked with community health workers, who could connect them to local resources such as walking clubs, share resources about coping skills, and provide psychosocial support. Utilizing community members to support education efforts is a well-established method of patient education in the realm of public health to promote community engagement (Tang et al., 2014; Kangovi et al., 2017; Kim et al., 2016). Extending training of introductory pain management techniques to community health workers and other influential community members could provide meaningful support to rural adults with unmet and/or chronic pain management needs.

Further, rural Missourian’s inclination toward medical minimization warrants clinical attention. Although medical minimization is not inherently problematic, it can potentially lead individuals to reject evidence-based, high-benefit care (Scherer et al., 2020). Thus, to accommodate the inclination toward medical minimization among rural adults, culturally responsive medical approaches to pain management should foster clinician-patient relationships rooted in an understanding of shared rural values, emphasizing the role of pain management in maintaining or regaining baseline function and supporting patient autonomy through home-based, non-pharmacological strategies.

### 4.2. Study limitations

Several study limitations should be considered. First, in focusing specifically on the perspectives of rural adults in one state, we were able to contextualize our study in geographically and culturally specific realities that led to a rich understanding of participants’ experiences and views. However, consistent with a qualitative research approach (Creswell and Poth, 2025), we make no claims regarding the transferability of our findings to other geographic and/or cultural contexts. Second, because our sample reflected the racially and ethnically homogeneous composition of the counties from which participants were recruited, which are approximately 90 % non-Hispanic White, (Missouri Department of Health & Senior Services, 2023) it is unlikely that our study adequately captured the perspectives of racial and ethnic minorities living in rural Missouri. Third, our recruitment strategies (i.e., email, social media) and the conduct of interviews via telephone may have limited participation among individuals with constrained access to technology or telecommunications. Fourth, we did not explore potential gender differences in participants’ conceptualizations of pain and attitudes toward pain management, as this focused analysis was not the emphasis of the study. However, given extensive research demonstrating gendered experiences of pain and pain treatment (Osborne and Davis, 2022), we encourage future research examining gender-related patterns in rural populations to better understand these dynamics. Finally, participation in the study was limited to one interview, which precluded techniques such as prolonged engagement and data triangulation, which would have enriched the methodological rigor of the study had they been employed (Henderson and Rheault, 2004).

## 5. Conclusion

This study examined rural Missourians’ conceptualization of pain and their attitudes toward pharmacological and non-pharmacological pain management. Participants framed pain primarily as a limitation on daily life, with tolerance linked to toughness and treatment often perceived as weakness. Pharmacological management was often met with stigma, fears of addiction or loss of control, and general aversion, while non-pharmacological strategies, particularly natural and preventive approaches, were strongly favored and aligned with cultural ideals of self-sufficiency and autonomy. Taken together, these findings underscore the need for culturally responsive approaches to pain care that not only resonate with place-based rural values but also inform broader conversations about health, culture, and care in rural communities.

## Figures and Tables

**Table 1:** Participant demographics.

	Overall (*n* = 25)

**Age Range** (n, %)	
30–39	4 (16 %)
40–49	5 (20 %)
50–59	3 (12 %)
60–69	9 (36 %)
70–79	4 (16 %)
**Sex**	
Male	4 (16 %)
Female	21(84 %)
**Race**	
Asian	1 (4 %)
Black/African American	2 (8 %)
White	22 (88 %)
**Relationship Status**	
Single, never married	4 (16 %)
Married or engaged	17 (68 %)
Divorced or separated	2 (8 %)
Widowed	2 (8 %)
**Highest Formal Education**	
High school or equivalent	5 (20 %)
Some college/trade school	7 (28 %)
Undergraduate degree	4 (16 %)
Graduate degree	9 (36 %)

**Table 2:** Themes describing rural Missourians’ conceptualizations of pain and attitudes toward pain management.

Category	Theme	Definition/Description

Conceptualizing Pain	Pain as a limitation	Participants experienced pain as interfering with daily activities, productivity, and personal agency; pain is viewed as a nuisance or obstacle.
	Pain as weakness	Pain tolerance is equated with personal strength; acknowledging or treating pain is often seen as a sign of weakness.
Attitudes Toward Pharmacological Pain Management	Social stigma	Perceived negative judgments from the community toward individuals using opioid or other pain medications, particularly for non-terminal pain.
	Fear of losing control	Concerns that taking pain medication could lead to addiction or impair cognitive/physical functioning, threatening personal autonomy.
	General aversion to medication	Broad dislike or avoidance of medication, often driven by perceived side effects, preference for natural remedies, or past negative experiences.
Attitudes Toward Non-Pharmacological Pain Management	Preference for natural interventions	Strong interest in non-drug approaches to managing pain, including physical activity, stretching, yoga, meditation, ice, vitamins/minerals, or other natural remedies.
	Emphasis on prevention	Focus on maintaining health to prevent pain, such as regular exercise, healthy diet, stress management, and proactive self-care.

## Data Availability

The authors do not have permission to share data.
